# Gastric Cancer or Plasmacytoma in a Seemingly Well-Controlled Multiple Myeloma Patient?

**DOI:** 10.4274/tjh.galenos.2021.2020.0645

**Published:** 2021-06-01

**Authors:** Wanlu Ma, Boju Pan, Lu Zhang

**Affiliations:** 1 Peking Union Medical College Hospital, Chinese Academy of Medical Sciences and Peking Union Medical College, Department of Endocrinology, Beijing, China; 2 Peking Union Medical College Hospital, Chinese Academy of Medical Sciences and Peking Union Medical College, Department of Pathology, Beijing, China; 3 Peking Union Medical College Hospital, Chinese Academy of Medical Sciences and Peking Union Medical College,Department of Hematology, Beijing, China

**Keywords:** Multiple myeloma, Gastrointestinal plasmacytoma, Gastric hemorrhage

## To the Editor,

**Figure 1:**
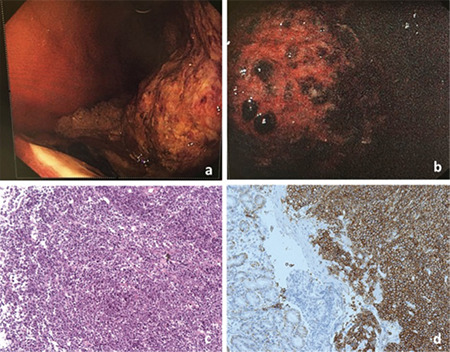
**a, b)** Gastroscopy showed a giant tumor in the stomach cavity, extending from the fundus and stomach to the stomach horn and anterior wall of the gastric antrum, with ulcers on the surface, covered with moss-like growth and bloody scab. **c, d)** Pathology of the body of the stomach confirmed plasmacytoma (plasmoblast type). **c)** Hematoxylin and eosin staining magnified 100 times. **d)** CD138 immunohistochemical staining magnified 100 times.

An 89-year-old man complained of hip joint pain accompanied by fatigue, anorexia, nausea, and vomiting for 1 year. Lab assessments showed anemia with hemoglobin (Hgb) of 68 g/L. Liver and renal functions and serum calcium were normal. Computed tomography (CT) and positron emission-computer tomography exhibited multiple osteolytic lesions throughout the whole body. Serum protein electrophoresis showed elevated M protein (31.4 g/L). Serum and urine immunofixation electrophoresis revealed strong positive immunoglobin G λ. Urine light chain (24 hours) was negative (below the lower limit of detection). A bone marrow smear showed myeloma cells at a rate of 12.5%. Fluorescence in situ chromosome hybridization did not detect 1q21+, 17p-, t(14;16), t(4;14), or t(11;14). Multiple myeloma (MM) (DS IIIA, ISS II, R-ISS II) was diagnosed with albumin of 22 g/L, serum β2-microglobulin of 5.05 mg/L, and lactate dehydrogenase of 308 U/L (normal range: 97-270). An LD regimen (lenalidomide at 25 mg per day for 21 days, dexamethasone at 10 mg per week) was administered for 8 months. His condition improved with normal Hgb (120 g/L). Serum M protein gradually decreased to 0.3 g/L for 7 months before he suffered from hematemesis and melena. Thoracoabdominal CT showed irregularly thickened gastric wall of the sinuses and greater curvature of the body of the stomach. Gastroscopy showed a giant tumor extending from the fundus to the stomach horn and anterior wall of the gastric antrum, which resembled gastric cancer (Figures 1a and 1b). However, to our surprise, pathology revealed plasmacytoma (plasmoblast type), which suggested disease progression despite well-controlled serum M protein (2.2 g/L) (Figures 1c and 1d). Urine light chain also reached 476 mg/24 h despite a negative urine light chain level at baseline. The patient progressed rapidly and his general condition worsened as he suffered from not only gastric hemorrhage but also pulmonary infection. Bortezomib was intended to be given but, considering his older age, extremely poor condition, and the wishes of the patient and his family, palliative care was given. Unfortunately, the patient died of pulmonary infection 10 days later.

MM is a neoplastic proliferation of monoclonal plasma cells [1,2]. Gastrointestinal (GI) involvement is not common and GI hemorrhage is rarely reported [3]. Here we have presented a case of MM and a giant gastric tumor. We did not expect the tumor to be a plasmacytoma before pathology, mainly due to three reasons. First, involvement of the GI system is rare in MM [1]. Previous series suggested little or no GI involvement in autopsied MM patients [2]. A systematic review of 2584 MM patients identified only 24 cases (0.93%) with involvement of the GI system [1]. Among the 4 cases with stomach involvement, only 1 case showed symptoms of upper GI bleeding with a mass of 4x10 cm [1]. Second, this patient seemed to have well-controlled myeloma with decreased M protein compatible with a very good partial response (VGPR) [3] and normalization of hemoglobin. It would be even rarer if a patient suffered from a rare manifestation of myeloma with well-controlled disease. Third, MM could be accompanied by second primary malignancies and the reported rate (3.5%-4.52%) was higher than that of gastric involvement (0.93%) in MM patients [1,4].

However, pathology showed plasmacytoma, which indicated progression of MM [3]. Moreover, despite a negative 24-h urine light chain level at baseline and marked decrease of intact serum M protein compared to baseline (31.2 g/L decreased to 2.2 g/L), significantly elevated urine light chain levels also suggested disease progression. This phenomenon was termed “light chain escape,” and clinicians should examine urine light chain even in patients with normal 24-h urine light chain level at baseline [5].

Previous studies showed that extramedullary plasmacytoma at relapse had the worst prognosis (progression-free survival of 13.6 months and overall survival of 11.4 months) [6] and patients who progress within 18 months of initial therapy have particularly poor outcomes [7]. Considering the age, poor general condition, possible outcomes, and wishes of the patient and his family members, palliative care was given. Despite sufficient supportive treatment, the patient died soon.

We report a case of GI hemorrhage rarely seen in MM. Due to spatial heterogeneity, malignant cells at different anatomical locations may display different levels of sensitivity.
